# The effects of 5-HT on vascular endothelial dysfunction in patients with panic disorder

**DOI:** 10.3389/fcvm.2025.1632070

**Published:** 2025-08-20

**Authors:** Yueqi Feng, Xixi Li, Zijia Li, Xinyi He, Yanqing Tang, Wen Tian

**Affiliations:** ^1^ Department of Geriatrics, The First Hospital of China Medical University, Shenyang, Liaoning, China; ^2^ Department of Psychiatry, Shengjing Hospital of China Medical University, Shenyang, Liaoning, China

**Keywords:** panic disorder, 5-HT, selective serotonin reuptake inhibitors, vascular endothelial function, signaling pathways, cardiovascular diseases

## Abstract

Panic Disorder (PD) is a prevalent psychiatric condition characterized by recurrent episodes of acute severe anxiety. These episodes frequently present with symptoms that overlap with those of cardiovascular diseases (CVD), such as elevated blood pressure and chest pain. Despite the prevalence and impact of this comorbidity, the underlying mechanisms are not well understood and remain underexplored. This review synthesizes current understanding and recent findings on the role of 5-hydroxytryptamine (5-HT) in the intersection of PD and vascular dysfunction. 5-HT, a critical inhibitory neurotransmitter, has been implicated in the etiology of PD and linked to panic symptoms. This review underscores the importance of 5-HT in modulating vascular tone through its action on 5-HT1B and 5-HT2A receptors, influencing the production of nitric oxide (NO) and the subsequent vasomotor response. Furthermore, the impact of 5-HT system on platelet activation and aggregation adds another layer to the complex relationship between PD and CVD. Selective serotonin reuptake inhibitors (SSRIs) have shown promise in improving vascular endothelial function. However, the influence of SSRIs on CVD outcomes remains a controversial issue with conflicting findings from various studies. The review also highlights the role of the PI3K/Akt/eNOS signaling pathway in 5-HT's influence on vascular endothelial function. In conclusion, the intricate relationship between PD, 5-HT, and vascular endothelial function warrants further investigation. A deeper understanding of these mechanisms could lead to more effective treatments for PD and related CVD, ultimately improving patients’ mental health and cardiovascular outcomes.

## 1 Introduction

Panic Disorder (PD) is a prevalent psychiatric condition characterized by recurrent episodes of acute, severe anxiety, known as panic attacks. These episodes can emerge independently or coexist with other mental health disorders, such as agoraphobia and depression. Panic attacks (PAs) frequently present with symptoms that overlap with those of cardiovascular diseases (CVD), such as elevated blood pressure, palpitations, and chest pain. Individuals experiencing a panic attack may be scared of dying from a heart attack during the episode. Studies have revealed that over 20% of patients undergoing cardiac emergency treatment do not have heart disease but are suffering from PD (1–3). Typically, these patients maintain a level of skepticism even after cardiac examinations yield negative results.

A wealth of epidemiological evidence has demonstrated a robust association between PD and CVD caused by vascular endothelial dysfunction. This includes conditions such as coronary heart disease (e.g., myocardial infarction and angina) and hypertension (2, 4–6). However, the precise mechanisms underlying this association remain unclear. This article will focus on the relationship between PD and endothelial dysfunction, as well as explore the potential mechanisms involved.

## 2 Panic disorder and its biological markers

The definition of PD has undergone several revisions in the Diagnostic and Statistical Manual of Mental Disorders (DSM) in the United States. The fifth edition, DSM-5, classifies PD as a subtype of anxiety disorder (7).

### 2.1 Clinical features of PD

PD is characterized by recurrent, unexpected PAs, which may be accompanied by symptoms such as fear of subsequent PAs, worry about the potential impact or consequences of these attacks, and significant behavioral changes related to the attacks. It is important to note that fear and panic behaviors are considered protective states, and PAs are automatic responses to protect individuals from life-threatening situations (8). The primary symptoms of PAs include cardiovascular symptoms (e.g., chest pain, increased blood pressure), autonomic nervous system responses (e.g., accelerated heartbeat, sweating), respiratory difficulties (e.g., dyspnea, chest tightness, a sensation of suffocation), and cognitive symptoms (e.g., depersonalization, fear of losing control, fear of death) (9). Among these, cardiac-related symptoms are most frequently reported (8).

### 2.2 5-HT as a biomarker for PD

Biomarkers are measurable indicators of normal biological processes, pathological processes, or responses to an intervention. Numerous studies have identified various biomarkers that reflect PD, including 5-HT and cortisol levels, and heart rate variability. The structural changes of the amygdala or hippocampus and cerebral blood flow in the left occipital cortex are considered to be potential markers for PD (10–14).

Serotonin, also known as 5-hydroxytryptamine (5-HT), is an endogenous monoamine and a critical inhibitory neurotransmitter widely distributed in the central and peripheral nervous systems. It plays a vital role in regulating emotions, cognition, anxiety, learning, memory, and sleep (15).

The hypothesis of serotonin system imbalance is widely recognized in the etiology of PD. Carbon dioxide inhalation-induced panic attacks and neuroimaging studies have shown that the serotonin system is closely related to panic symptoms (16). The mechanism may be that serotonin inhibits anxiety-specific adaptive behavior, has the effect of maintaining alertness, and controls anxiety, and serotonin can inhibit the dorsal periaqueductal gray (PAG) structure related to fear response behavior in PD (17, 18). Consequently, PD patients often exhibit reduced 5-HT levels in the brain (11). Abnormally increased serotonin turnover rates have been observed in PD patients (19), and the serotonin transporter (SERT) encoding gene SLC6A4 was demonstrated as a promising diagnostic gene for PD (20). Peripherally, studies have confirmed lower serum serotonin levels in PD patients compared to controls (21), and other studies have shown increased 5-HT uptake by platelets in these patients (22, 23). The cause of this change in peripheral 5-HT levels, whether due to reduced synthesis or increased reuptake or degradation, remains unclear. Clinically, selective 5-HT reuptake inhibitors (SSRIs), such as fluoxetine, paroxetine, and sertraline, are first-line treatments for PD (24). They work by inhibiting the 5-HT transporter (SERT) to reduce 5-HT reuptake, thereby increasing the concentrations in the synaptic cleft and restoring 5-HT balance in the brain.

## 3 The impact of Pd on vascular endothelial function

### 3.1 Physiological structure and function of vascular endothelial cells

Vascular endothelial cells (ECs) form a single layer lining the interior walls of arteries, veins, and capillaries. The primary function of ECs is their barrier role, forming an inner lamina of blood vessels that restricts the passage of large molecules into the vessel wall (25). ECs also regulate vascular tone by sensing changes in blood flow dynamics (blood shear stress) and releasing endothelium-dependent vasodilatory and vasoconstrictive factors. Nitric oxide (NO) is the principal vasodilatory factor released by ECs (26), and they also produce prostacyclin (PGI2) to relax vascular smooth muscle (25). Conversely, ECs can release vasoconstrictive factors such as endothelin-1 (ET-1), angiotensin II (AngII), thromboxane A2, prostaglandin H2 (PGH2), and reactive oxygen species (ROS) (27, 28). Under normal conditions, the balance of vasoactive factors released by ECs maintains vascular tone and vascular homeostasis (29).

Vascular endothelial dysfunction can occur due to various unhealthy lifestyles and physicochemical factors, such as oxidative stress and inflammation. This dysfunction is characterized by a decrease of NO production and impaired endothelial barrier function. Reduced NO can impair vasodilation, and the disruption of endothelial barrier function can lead to the influx of low-density lipoprotein (LDL) into the arterial wall, the adhesion of monocytes and platelets, and the localized release of cytokines and growth factors. This can cause the migration, proliferation, and phenotypic transformation of vascular smooth muscle cells (VSMCs), resulting in the development of atherosclerosis (30). Vascular endothelial dysfunction is thus a contributing factor in the progression of various cardiovascular diseases, including hypertension, atherosclerosis, and acute coronary syndrome (31, 32).

### 3.2 PD and vascular endothelial dysfunction

PD or PAs are often associated with symptoms like chest pain and increased blood pressure. Given the role of endothelium in regulating vascular tension, it is hypothesized that there may be a close relationship between PD and vascular endothelial dysfunction.

Flow-mediated dilatation (FMD) is the clinical standard for evaluating vascular endothelial function. Clinical studies found that psychosocial stress could reduce FMD response in healthy men (33), and anxiety disorders are significantly associated with coronary endothelial dysfunction (CED) in women presenting with chest pain and nonobstructive coronary artery disease by using invasive coronary reactivity testing (34).

Additionally, PD patients exhibit changes in inflammatory factor concentrations (35) and platelet activation (36), which may contribute to the development of CVD. A large-scale study demonstrated a positive correlation between PD and the incidence and mortality of CVD (37). Furthermore, the prevalence of PD is higher among patients with coronary heart disease, chronic heart failure, and hypertension compared to the general population (4, 38). PD is also linked to increased arterial stiffness (39), which can adversely affect cardiovascular health and endothelial function. A meta-analysis of 12 studies involving 58,111 cases of sudden cardiac events indicated that PD is independently correlated with the incidence of coronary heart disease, myocardial infarction (MI), and major adverse cardiovascular events (MACE) (40).

As mentioned above, apart from serotonergic mechanisms, PD is linked to increased sympathetic nervous system activity, systemic inflammation, and elevated levels of stress hormones like cortisol and catecholamines (19, 35). These factors can promote endothelial dysfunction through mechanisms including oxidative stress, reduced nitric oxide bioavailability, and vascular inflammation (34). Therefore, vascular changes in PD likely involve several interacting pathways.

## 4 The role of 5-HT in vascular endothelial function

As previously discussed, the role of 5-HT as a biomarker for PD has been widely confirmed. However, research on the role of 5-HT in the impact of PD on vascular endothelial function is currently limited.

### 4.1 Synthesis and metabolism of 5-HT

**Figure 1:**
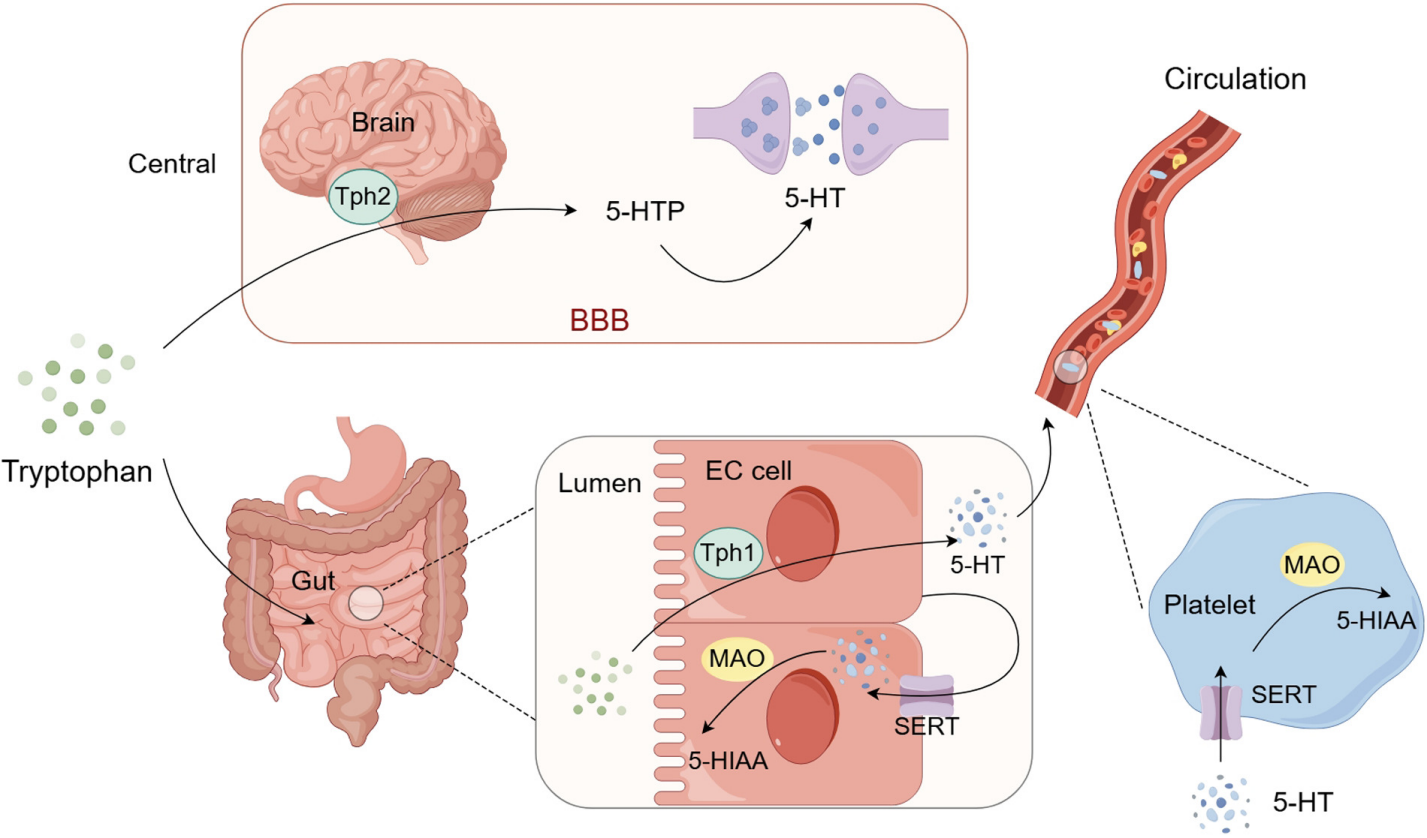
Schematic representation of 5-HT synthesis and metabolism. Tryptophan that enters the brain is synthesized into 5-HT by Tph2 and released into the synaptic cleft to exert its effects. Tryptophan that enters the gut lumen is synthesized into 5-HTby Tph1 in enterochromaffin cells. A small portion of this peripheral 5-HT is reuptaked back into the cells by the SERT of the enterochromaffin cells while the majority enters the blood circulation and is stored in platelets. When platelets are activated, 5-HT can be released and then exert its effects. Intracellular 5-HT can be metabolized by monoamine oxidase (MAO) into 5-HIAA and excreted in the urine. BBB: blood-brain barrier; Tph1: tryptophan hydroxylase 1; Tph2: tryptophan hydroxylase 2; SERT: 5-HT transporter; MAO: monoamine oxidase; 5-HTP: 5-hydroxytryptophan; 5-HIAA: 5-hydroxyindoleacetic acid. (By Figdraw.)

Due to the presence of the blood-brain barrier, the synthesis of 5-HT is typically divided into central and peripheral systems. Once tryptophan enters the body, it can be converted into 5-HT by tryptophan hydroxylase (Tph), with over 90% of 5-HT being synthesized by Tph1 in enterochromaffin cells of the intestine, and the remainder by Tph2 in the serotonergic neurons of the brainstem's raphe nuclei. Excess tryptophan is metabolized in the liver and excreted into the bloodstream (Figure 1).

Regarding the metabolism of 5-HT, a small portion of centrally synthesized 5-HT is released into the synaptic cleft to act on various regions of the brain, while the surplus is reabsorbed by the 5-HT transporter (SERT) on the presynaptic membrane and metabolized by monoamine oxidase (MAO) into 5-hydroxyindoleacetic acid (5-HIAA), which are then excreted in urine. The 5-HT synthesized in enterochromaffin cells has two main fates: 1. a significant portion is released into the intestinal lamina propria, where some are reabsorbed by SERT on the enterochromaffin cell membrane and metabolized by MAO on the outer mitochondrial membrane of intestinal epithelial cells into 5-HIAA; the rest enters the blood circulation through the capillary bed of the intestinal mucosa, where most is reabsorbed by SERT on platelets and stored in dense granules. Ultimately, a large amount of 5-HT stored in platelets can be transported throughout the bloodstream, and when platelets are activated, they can release their granule contents, including 5-HT, binding to various receptors and exerting effects such as regulating vascular tone, permeability and inducing platelet activation and aggregation (41, 42); 2. a small portion of 5-HT is released into the intestinal lumen through the apical membrane, mixed with chyme, and eventually excreted with feces (Figure 1).

Under normal conditions, only about 2% of peripheral 5-HT is free in the bloodstream, while approximately 98% is stored in platelets. The freely circulating fraction determines immediate bioavailability (43). Free 5-HT produces various effects by binding to 5-HT receptors on the surface of various cell membranes. Platelets can release stored 5-HT rapidly upon activation triggered by vascular injury, thrombin, collagen, or shear stress (44). SERT is not only distributed on the presynaptic membrane of nerve terminals, but also expressed in platelets, gastrointestinal chromaffin cell membranes, vascular endothelial cells, myocardium, and kidneys. As a clearance tool for 5-HT, SERT can terminate its action by absorbing 5-HT.

### 4.2 Regulation of vascular tone by 5-HT

Peripherally distributed 5-HT can have complex and even biphasic effects (vasoconstriction/vasodilation) on peripheral blood vessels. The complex biological functions of 5-HT are related to its multiple receptor subtypes. To date, seven major classes of 5-HT receptors have been identified, namely 5-HT1-7 receptors, with a total of 14 receptor subtypes (45).

5-HT acts on several receptor types in the vasculature. The 5-HT1B receptor is expressed on ECs, is linked to Gi/o proteins, and mediates vasodilation through NO production via the PI3K/Akt/eNOS pathway. In contrast, the 5-HT2A receptor on VSMCs causes vasoconstriction through phospholipase C activation and calcium signaling. These receptors couple to Gq proteins, activating phospholipase C (PLC). PLC hydrolyzes phosphatidylinositol 4,5-bisphosphate (PIP2) into diacylglycerol (DAG) and inositol trisphosphate (IP3). IP3 then triggers calcium release from the sarcoplasmic reticulum, raising intracellular calcium levels and promoting smooth muscle contraction (43, 45). Therefore, selectively blocking the 5-HT2A receptor and selectively activating the 5-HT1B receptor are considered potential mechanisms for vascular protection (46, 47). Other receptors, such as 5-HT2B and 5-HT7, may also influence vascular tone in specific regions. These receptors activate downstream signaling cascades that regulate vascular tone and endothelial function (43). Sapogrelate, an antagonist of the 5-HT2A receptor, can indirectly enhance the function of the 5-HT1B receptor on ECs by inhibiting the 5-HT2A receptor on VSMCs, thereby increasing NO production and vasodilatory response (48–50).

Meanwhile, the vasoactive effects of 5-HT on blood vessels also depend on the integrity of the endothelium. When the endothelium is intact, 5-HT acts on receptors of ECs to induce vasodilation, whereas when the endothelium is injured, 5-HT may directly act on receptors of VSMCs to induce vasoconstriction (51). In addition to vascular ECs, the 5-HT1B receptor is also distributed on VSMCs, and contrary to its EC-derived vasodilatory effect, it mediates vasoconstriction in VSMCs. The function mediated by the 5-HT1B receptor was analyzed using arteries with intact endothelium and with denuded endothelium. It was found that the presence of intact endothelium hinders the 5-HT1B receptor-mediated contraction of VSMCs (52). Rat-based experiments have confirmed that when the endothelium is impaired, 5-HT can directly act on the 5-HT1B receptor of VSMCs, inducing vasospasm (53). The findings underscore the critical role of intact endothelium in maintaining the equilibrium of 5-HT's vasomotor effects.

### 4.3 5-HT promotes platelet activation and aggregation

Platelets, lacking tryptophan hydroxylase (Tph), do not have the capability to synthesize 5-HTserotonin. Under physiological conditions, platelets act as carriers of 5-HTserotonin, storing it in their dense granules. Upon activation by stimuli such as thrombin, collagen, or shear stress, platelets release 5-HT into the local environment (44). During platelet activation, 5-HTserotonin is secreted from these granules and acts on the 5-HT2A receptors on the platelet surface, accelerating platelet activation and aggregation (54). This receptor activation increases intracellular calcium levels, leading to shape change, granule secretion, and activation of integrin αIIbβ3, which is essential for fibrinogen binding and platelet-platelet interactions. 5-HT can thereby act as a positive feedback mediator in thrombosis (54). Moreover, studies on serotonylation have suggested that serotonylation of small GTPases can trigger the release of platelet α-granules (55). Ultrastructural analysis has also observed that 5-HT serotonin can induce the formation of pseudopodia in platelets (56). Activated platelets can further secrete other pro-coagulant substances through exocytoses, such as fibrinogen, platelet-derived growth factor, and platelet factor 4 from α-granules (57), which are released into the bloodstream, leading to secondary platelet aggregation and an increased risk of thrombosis.

Additionally, when platelets are activated, they can induce the production of vasoconstrictors such as thromboxane A2 (TXA2), which may also be part of the reason why 5-HT can promote vasoconstriction. Elevated platelet activation in PD may contribute to a higher risk of endothelial dysfunction and vascular events.

## 5 PD modulates vascular endothelial function via 5-HT mechanisms

Although central 5-HT plays a primary role in the pathophysiology of PD, peripheral free 5-HT, despite its low concentration, can exert significant biological effects, particularly in the regulation of vascular tone. As the majority of peripheral 5-HT is stored in platelets, platelet aggregation and activation under conditions such as endothelial injury or inflammation can rapidly alter circulating 5-HT levels, enabling it to act through high-affinity receptor binding (43, 58, 59). As mentioned above, PD is characterized by decreased peripheral 5-HT levels. It is supposed that during PAs, 5-HT predominantly targets the 5-HT1B receptors on ECs, and its diminished levels result in attenuated NO synthesis via the 5-HT1B receptor pathway, consequently lessening the extent of vasodilation. Moreover, patients with PD often exhibit platelet activation and endothelial dysfunction, which may lead to increased release of serotonin from platelets and further enhance the role of peripheral 5-HT in regulating vascular tone in this disorder.

### 5.1 The influence of SSRIs on vascular endothelial function

The SERT is mainly distributed on the presynaptic membrane and various types of non-neuronal cells, including platelets, ECs, and myocardial cells. The levels of 5-HT and the timing of its transmission in the synaptic cleft and peripheral bloodstream are meticulously regulated by the reuptake of free 5-HT through the SERT. The development of selective 5-HT reuptake inhibitors (SSRIs) is centered on targeting this SERT mechanism, representing the first-line therapeutic approach for PD.

Regarding the influence of SSRIs on vascular endothelial function, some studies have found that during treatment with escitalopram, the levels of biomarkers of vascular endothelial dysfunction, such as soluble von Willebrand factor (sVWF) and vascular cell adhesion molecule-1(VCAM-1), gradually decrease in patients with major depression (60). A study has confirmed the impact of psychological stress on the activation of pathways related to vascular endothelial growth factor (VEGF), which affects endothelial cells, and impairs neovascularization following ischemia, and fluoxetine may offer a potential therapeutic benefit (61). A recent meta-analysis has demonstrated that treatment with SSRIs can lead to a significant improvement in FMD, suggesting that SSRIs may have a beneficial impact on vascular endothelial function beyond their effects on mood and anxiety disorders (62).

The influence of SSRIs on CVD, particularly thrombosis, is a subject of debate with two divergent perspectives. Some researchers have found that SSRIs can have a protective effect against MI through cohort studies and meta-analysis (63, 64), which may be related to SSRIs reducing the storage of 5-HT in platelets, thereby reducing platelet activation. Kim et al. followed up 300 patients with acute coronary syndrome and depression for 8 years and found that the incidence of MI in patients taking escitalopram was significantly lower than in those taking a placebo (65). However, some other studies suggested that SSRIs may exacerbate the deterioration of CVD. In an experiment with atherosclerotic mice, it was found that intake of fluoxetine can deplete 5-HT in platelets, induce a significant reduction in serum 5-HT, and promote the formation of atherosclerotic plaques by enhancing the activation of adhesion molecules (66). A recent study also found that escitalopram treatment can increase the degree of myocardial fibrosis in mice with MI combined with chronic mild stress (67). The findings indicate that a cautious approach is warranted when prescribing SSRIs to patients with concurrent cardiovascular and psychiatric conditions.

### 5.2 The modulation of vascular endothelium function by 5-HT via the Pi3k/Akt/eNOS signaling pathway

Extensive clinical researched have substantiated the correlation between CVD and mental health disorders, with 5-HT potentially serving as a common pathological mechanism. However, a comprehensive understanding of the serotonergic pathway—from its origins to its terminal effects—and its precise role in modulating vascular endothelial function remains elusive. Thus, meticulously exploring the complex interplay among 5-HT, its signaling pathways, and CVD is a promising frontier for progress in psycho-cardiology.

**Figure 2:**
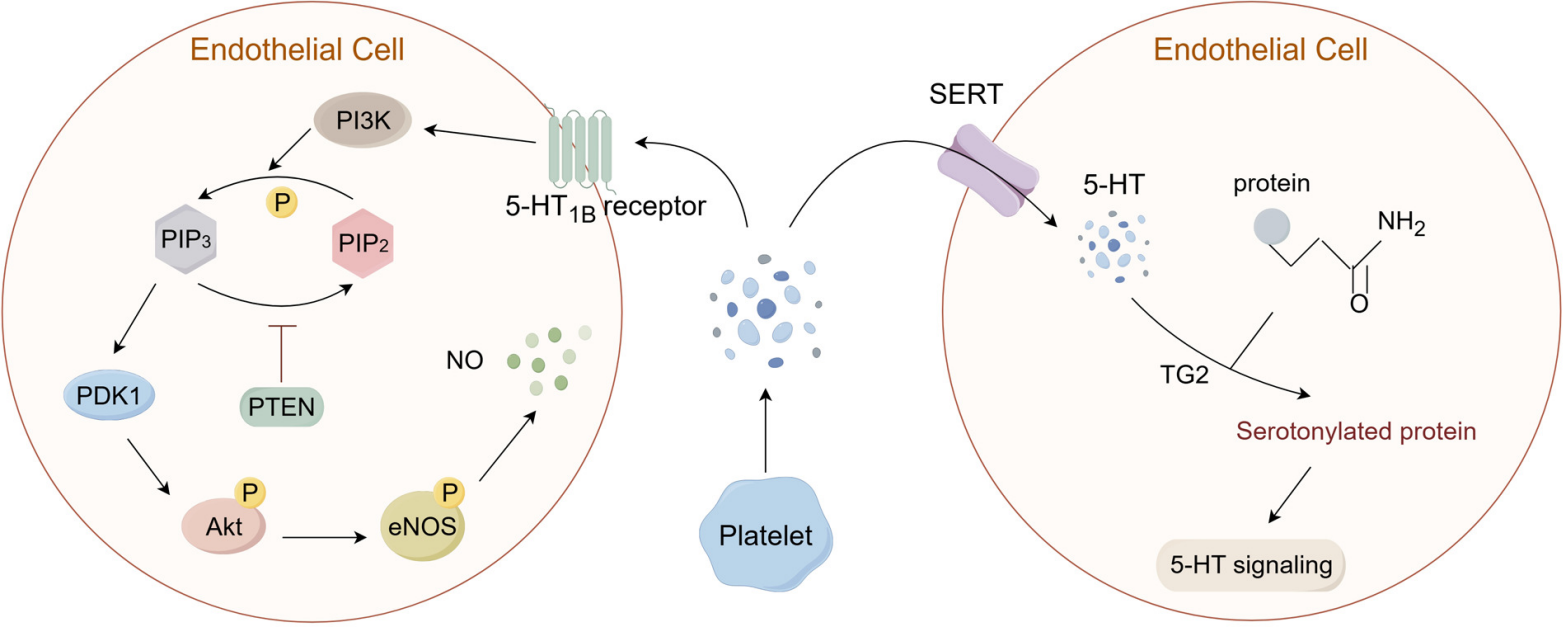
5-HT can transmit signals through two pathways: G protein-coupled receptors (GPCRs) and serotonylation. (1) In ECs, 5-HT can bind to the 5-HT1B receptor, leading to the activation of the PI3K/Akt pathway and the phosphorylation of eNOS, which mediates the generation of NO. (2) In addition, 5-HT that is reuptaked into the cell via SERT can combine with protein substrates containing glutamine residues under the catalysis of TG2, causing serotonylation of the substrates and the activation of related signaling pathways. PI3K: phosphoinositol-3 kinase; PIP2: phosphatidylinositol-4,5-bisphosphate; PIP3: phosphatidylinositol-3,4,5-trisphosphate; PTEN: phosphatase and tensin homolog; PDK1: 3-phosphoinositide-dependent protein kinase-1; Akt: Protein Kinase B; eNOS: endothelial nitric oxide synthase; NO: nitric oxide; TG2: transglutaminases 2. (By Figdraw.).

Studies have confirmed that 5-HT can stimulate angiogenesis by activating Protein Kinase B (Akt) in ECs (68), and fluoxetine can increase the expression of Akt, protecting vascular endothelium in a mouse model of psychological stress (61). It was discovered that the physiological levels of 5-HT are capable of inducing the phosphorylation of PI3K and Akt via the 5-HT1B receptor in cultured primary ECs (69). Activation of 5-HT1B receptors on ECs is linked to Gi/o proteins, which inhibit adenylate cyclase and activate the PI3K/Akt pathway. PI3K phosphorylates PIP2 to generate PIP3, which attracts PDK1 to the membrane. PDK1 then phosphorylates Akt. This leads to phosphorylation of endothelial nitric oxide synthase (eNOS), increasing nitric oxide (NO) production (Figure 2) (70–72). Based on previous research, the PI3K/Akt signaling cascade may predominantly influence three critical aspects within endothelial cells, i.e., cellular survival and apoptosis, the inflammatory response, and oxidative stress. Activation of the PI3K/Akt/eNOS pathway can induce the release of NO from ECs, which induces vasodilation and exhibits vascular protection and is key to maintaining vascular homeostasis (73). NO diffuses into adjacent VSMCs, stimulates soluble guanylate cyclase (sGC), elevates cyclic GMP levels, and promotes smooth muscle relaxation, contributing to vasodilation (74). Conversely, the inhibition of the PI3K/Akt pathway can diminish the phosphorylation of eNOS, consequently leading to a decrease in NO synthesis (75). Furthermore, NO possesses the ability to inhibit reactive oxygen species (ROS), thereby mitigating the oxidative stress associated with vascular endothelial dysfunction (76).

### 5.3 Intracellular 5-HT can mediate the serotonylation of substrates

The peripheral 5-HT exerts its effects by binding to 5-HTR on the cell membrane, which has been widely recognized. However, 5-HT can also exert intracellular effects through a process known as serotonylation. Although these pathways operate via distinct processes, they collectively influence vascular tone and endothelial behavior (Figure 2). In recent years, it has been discovered that the 5-HT taken up into the cell by SERT can also exert its effects (77). Over 60 years ago, researchers discovered that 5-HT could covalently bind to certain proteins under the action of transglutaminases (TG), which is a new type of post-translational modification (PTM) known as serotonylation. However, it was only in recent years that the mechanisms related to serotonylation have gradually been elucidated (78–80). TG is a multifunctional enzyme composed of eight isoenzymes, known as factor XIII and TG1-7. Among them, TG2 is widely expressed in almost all tissues and participates in a variety of biological processes. TG2 has GTP-binding activity and Ca2+-dependent transglutaminase activity (81). Under the action of Ca2+, TG2 is activated and acts on its common substrates, including small GTPases such as RhoA, histones, and other proteins like SERCA2a, Akt (79, 82, 83). Catalyzing the binding of substrates with 5-HT, thereby causing constitutive activation of the substrate and degradation through an enhanced proteasome pathway (Figure 2) (84).

The Rho protein family, as substrates of TG2, often undergo serotonylation, which leads to the activation of related signaling pathways. Studies have found that in ECs, the RhoA/ROCK pathway can affect vascular endothelial function and the synthesis of nitric oxide (NO) (85, 86). The ROCK inhibitor fasudil can reduce the expression levels of endothelin-1(ET-1) and endothelin receptors (ETR), inhibit the proliferation of pulmonary arterial endothelial cells, and promote the generation of NO (87). Moreover, inhibiting the RhoA/ROCK pathway can also activate the PI3K/Akt pathway, leading to the phosphorylation of eNOS, playing a protective role in vascular spasm and endothelial dysfunction (88–90).

## 6 Conclusions

In the rapidly evolving modern society, individuals frequently confront substantial psychological pressures. PD, a prevalent mental health condition, is intricately linked to the onset and poor prognosis of CVD. Therefore, a thorough understanding of the interplay between PD and CVD, along with the underlying mechanisms, is crucial for the effective management of psycho-cardiac conditions. Such understanding is pivotal not only for reducing the prevalence and mortality rates of CVD but also for enhancing the overall quality of life for individuals living with PD.
